# Viruses participate in the organomineralization of travertines

**DOI:** 10.1038/s41598-023-38873-5

**Published:** 2023-07-19

**Authors:** Mirosław Słowakiewicz, Edoardo Perri, Ezher Tagliasacchi, Paweł Działak, Andrzej Borkowski, Michał Gradziński, Sándor Kele, Maurice E. Tucker

**Affiliations:** 1 Faculty of Geology, University of Warsaw, 02-089 Warsaw, Poland; 2 Dipartimento di Biologia Ecologia e Scienze della Terra, Università della Calabria, 87036 Rende, Italy; 3 Faculty of Engineering, Pamukkale University, 20160 Denizli, Turkey; 4 Faculty of Geology, Geophysics and Environmental Protection, AGH University of Science and Technology, 30-059 Kraków, Poland; 5 Institute of Geological Sciences, Jagiellonian University, 30-387 Kraków, Poland; 6 Research Centre for Astronomy and Earth Sciences, Institute for Geological and Geochemical Research, Budapest, 1112 Hungary; 7 CSFK, MTA Centre of Excellence, Budapest, 1121 Hungary; 8 School of Earth Sciences, University of Bristol, Bristol, BS8 1RJ UK

**Keywords:** Biogeochemistry, Biogeochemistry

## Abstract

Travertines, which precipitate from high temperature water saturated with calcium carbonate, are generally considered to be dominated by physico-chemical and microbial precipitates. Here, as an additional influence on organomineral formation, metagenomic data and microscopic analyses clearly demonstrate that highly diverse viral, bacterial and archaeal communities occur in the biofilms associated with several modern classic travertine sites in Europe and Asia, along with virus-like particles. Metagenomic analysis reveals that bacteriophages (bacterial viruses) containing icosahedral capsids and belonging to the Siphoviridae, Myoviridae and Podoviridae families are the most abundant of all viral strains, although the bacteriophage distribution does vary across the sampling sites. Icosahedral shapes of capsids are also the most frequently observed under the microscope, occurring as non-mineralized through to mineralized viruses and virus-like particles. Viruses are initially mineralized by Ca-Si amorphous precipitates with subordinate Mg and Al contents; these then alter to nanospheroids composed of Ca carbonate with minor silicate 80–300 nm in diameter. Understanding the roles of bacteriophages in modern carbonate-saturated settings and related organomineralization processes is critical for their broader inclusion in the geological record and ecosystem models.

## Introduction

The term ‘travertine’ was introduced by ancient Romans and is defined as a hard crystalline rock generally containing fine laminae precipitated from non-saline high-temperature (hydrothermal) waters^1^. Typically, such thermal waters are supersaturated with calcium carbonate and depending on the temperature are colonized by prokaryotes, i.e., various groups of bacteria including cyanobacteria, as well as fungi, algae, bryophytes and vascular plants^2^. As a result of its dense fabric, colour, strength and aesthetics, travertine has been a much-valued building material since at least 1500 B.C., but the Romans were the first to use it on a large scale^3^. Precipitation of travertine is commonly the result of abiotic processes (notably vigorous degassing of CO_2_) but biological influences are also involved such that some travertine deposits are almost entirely microbially mediated^4,5^. Carbonate precipitation in travertine systems is controlled by physico-chemical (CO_2_ degassing, mixing, evaporation) and biologically-induced and -influenced processes where microbial biofilms can play an important role in crystal nucleation^6^. The study of modern microbial mats has shown that viruses can be initially mineralized by amorphous magnesium silicates, which quickly alter to magnesium carbonate nanospheres (~ 80–200 nm in diameter)^7^. Thus, not only bacteria, including their cell walls, sheaths and extracellular polymeric substances [EPS]^8,9^ but also viruses^7,10–16^ have been suggested to be involved in organomineralization^9,17^, a term that includes biologically-influenced mineralization sensu^9,17^. However, the role of viruses participating or influencing calcium carbonate mineralization in travertines is essentially unknown due to the lack of virus metagenomic data and evidence of organominerals associated with the ubiquitous presence of viruses in such ecosystems.

Here, we document how viruses contribute to the microbial communities that colonize the active surface of mineral deposition and are interlayered with travertine precipitates from several travertine systems in Europe and western Asia. We also show how they influence precipitation, leading to the formation of minerals in travertine, along with the key roles played by bacteria and polysaccharide matrices^18^. The microscopic identification of mineralized viruses and virus-like particles (VLPs, nanospheres which retain a virus-like morphology sensu^7^), supplemented with the elemental composition of VLPs and viral metagenomic analyses, allow the identification of changes in morphology, abundance, distribution and degree of mineralization of viruses in thermal travertine systems occurring at different latitudes in Europe and Asia, leading to a new biosignature for modern and fossil travertine deposits.

## Results

### Temperature, pH and water chemistry

All biofilm samples were collected in the spring of 2020 and 2021 (Fig. S1). The temperature of the water precipitating all travertines studied varies from 14.6 °C (Bešeňová) to 67 °C (Egerszalók) with pH between 6.2 (Asinello, Bešeňová) and 7.4 (Terme di Saturnia) (Supplemental materials, Tables S1, S2). The chemical composition of the thermal waters from each sampling site (Supplemental materials, Table S3) chiefly represents a Ca-HCO_3_ saturated system with various admixtures of other major and minor elements depending on the source of the waters. The Langelier saturation indices with respect to calcite (LSI_CAL_) calculated for each site range from − 0.41 to 1.2.

### Microscopy

Three fluorescent dyes were used to analyse the organization of the tested samples of biofilms associated with travertine deposits. The biofilm samples differed mainly in terms of microbial richness and in the degree of association of microbial cells with the mineral particles (Supplemental materials, Figs. S2 and S3). Bacterial cells, cyanobacteria, algae and individual structures resembling pollen of higher plants were observed on microscope slides. Mineral structures were also visible and, depending on the stain, showed weak fluorescence from green to greenish-yellow, or non-fluorescent phases as in the samples from Bešeňová. After SYBR^®^ Gold staining, large numbers of bacteria-like particles and the much smaller VLPs can be seen. However, these particles cannot be clearly distinguished. SYPRO Tangerine staining showed the connection between biofilms and mineral particles particularly well. The cells of the microorganisms were intensely red in contrast to the mostly greenish mineral structures. Two sample groups could be distinguished: (i) the samples in which mineral particles were strongly associated with microorganisms (Karahayıt, Bath), including cyanobacterial cells (Bullicame, Pamukkale), and (ii) other samples where loosely associated cells accompanied the mineral forms.

**Figure 1:**
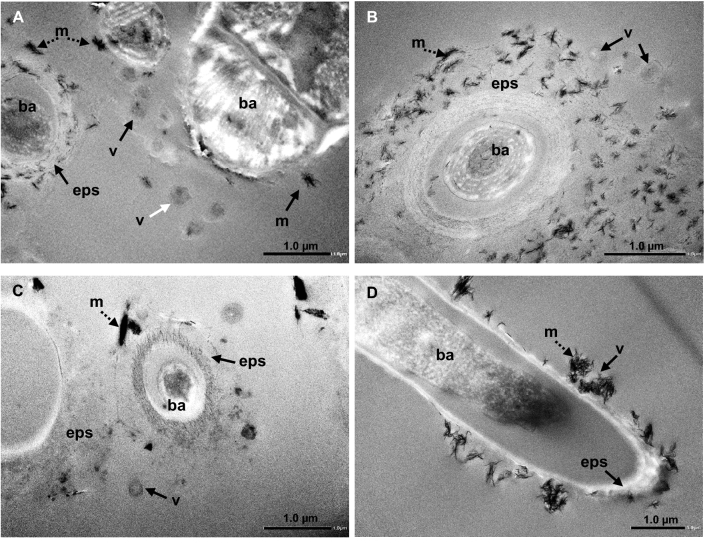
Representative TEM images of bacterial cells (ba), EPS (eps), virus-like particles (v), and neoformed minerals (m), in the biofilms covering the active depositional surface of travertines in the following sites: (**A**) Pamukkale; (**B**) Egerszalók; (**C**) Terme di Saturnia; (**D**) Bullicame. Note that the shape of the capsids (80–300 nm in size) varies from hexagonal (white arrows) to subspherical (dark arrows). Mineralization of organic substrates is variable and mainly in the EPS and on viruses with aggregates of nanocrystals (dashed arrows).

**Figure 2:**
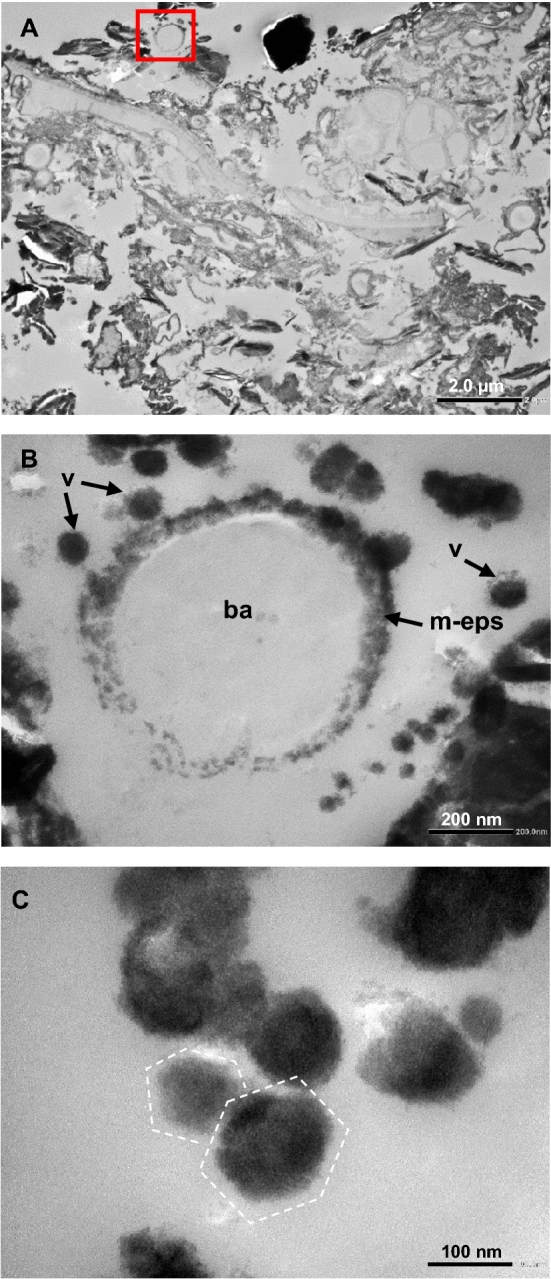
(**A**) TEM general view of the biofilm covering the active depositional surface of travertine at the Bešeňová site. Many sections of bacterial cells are visible together with EPS and minerals. The red square is the location of figure (**B**). (**B**) Close-up view of (**A**) in which a degraded bacterial cell (ba) is visible, surrounded by partially to fully mineralized EPS (m-eps). In the surrounding area several virus-like mineralized particles are also present (v). (**C**) Close-up view of virus-like mineralized particles, some of which have a hexagonal shape (dashed lines).

TEM observations of the biofilms associated with active travertine deposits, from all sampling sites, revealed abundant, 80–300 nm-sized particles, ranging from non-mineralized to fully mineralized, that exhibit hexagonal (or polyhedral) to subspherical (nanospheroidal) shapes (Fig. 1), consistent with a capsid-like morphology (Fig. 2). VLPs are commonly included in the EPS and some are attached to prokaryotic bacterial cell walls, as bacteriophages usually do. EPS can also be partially to totally mineralized themselves and among all the organic structures composing the biofilm, the EPS are the main sites where neo-formed minerals are nucleating and growing (Figs. 1 and 2).

**Figure 3:**
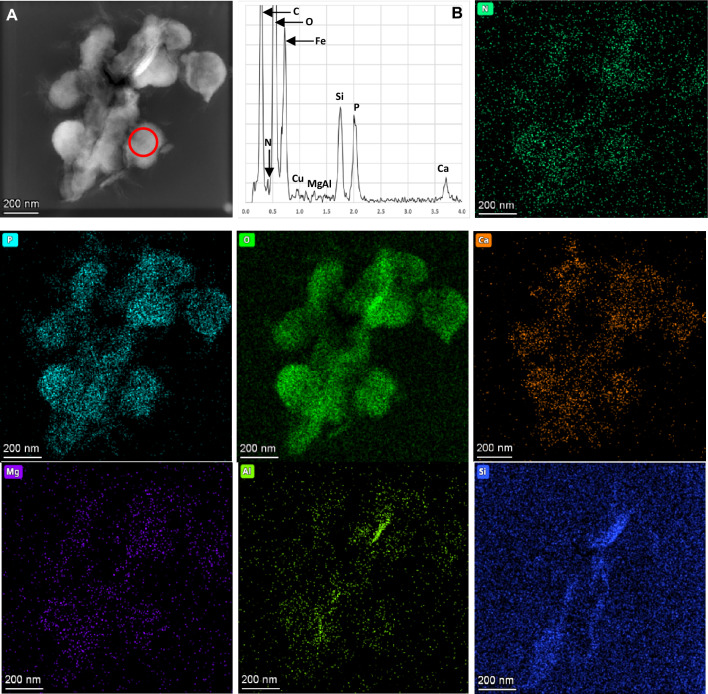
(**A**) Dark-Field STEM view of partially mineralized VLPs from the Bešeňová travertine. The red circle indicates the site of the EDS analysis reported in B; (**B**) EDS spectrum of the elements detected in a virus-like particle. Note that Cu and Fe in the EDS spectrum are due to contamination from the grid. Other images: Elemental compositional maps of mineralized VLPs in A. Note that VLPs are composed mainly of C, O, N, P and Ca, with minor amounts of Mg, Si and Al. Si and Al are, instead, more abundant in the central area where Si-Al-rich crystals are better-formed with respect to Ca-carbonate particles.

Elemental EDS analysis of the VLPs indicates that initial mineralization occurs with precursor Ca- and Si-rich phases, with low Mg and Al values, that eventually, as crystallization proceeds at these nucleation sites, evolve to well-formed Ca-carbonate crystals with minor silicates (Fig. 3). The same analysis shows that all VLPs, even if fully mineralized, contain P in a range of about 3.0–5.0 mol %, and 0.5–2.0 mol % N; both elements tend to be absent in well-developed minerals (Fig. 3). The molar N:P ratio measured in the studied VLPs averages 0.4.

### Metagenomics

**Figure 4:**
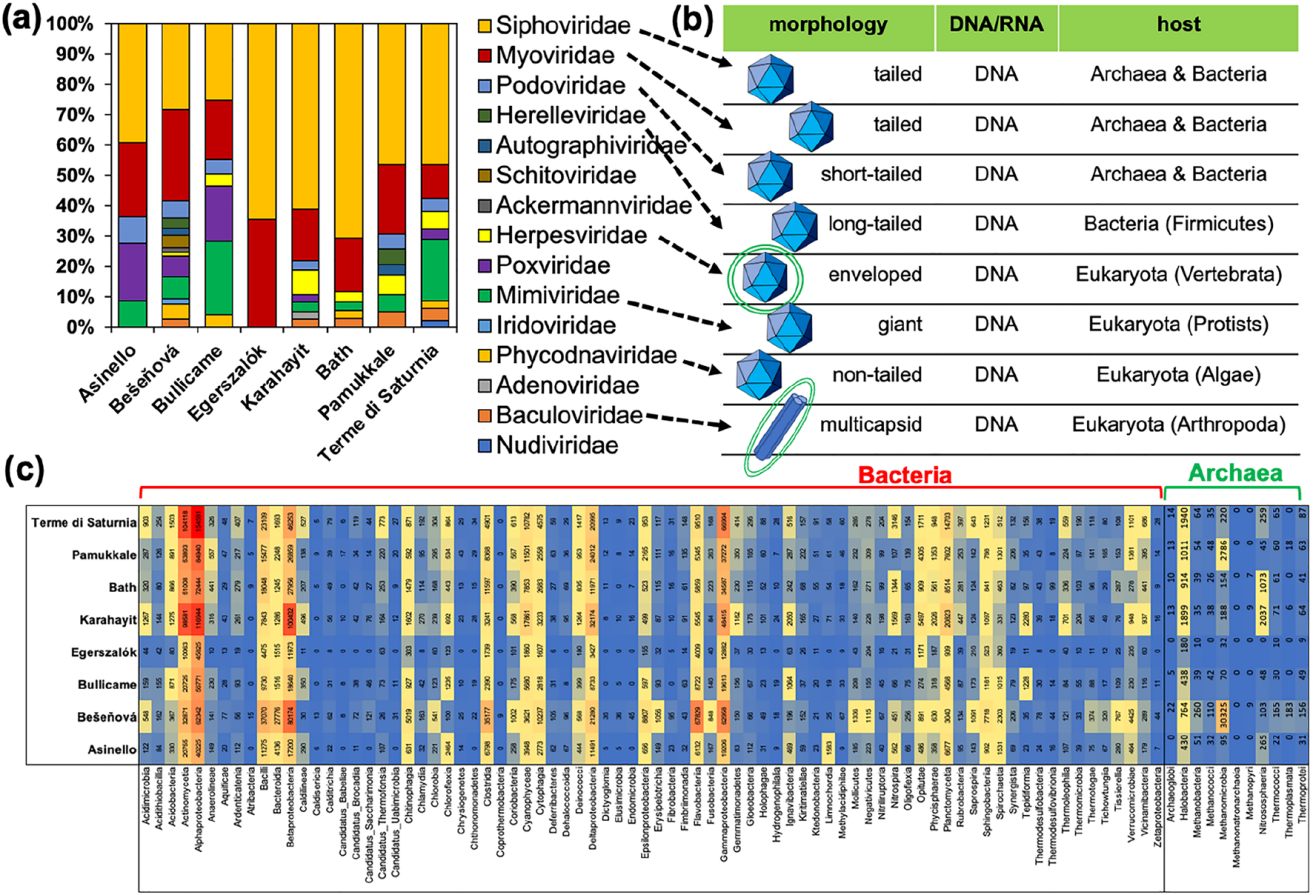
Metagenomic analyses of the collected biofilm samples. (**a**) Viral families detected in all samples. (**b**) Characteristic features of the main viral families. Icosahedral shape and lipid envelope are graphically marked. (**c**) Heatmap showing classes of bacteria and archaea. The numbers indicate the count of contigs. The colours indicate relative abundance.

The bioinformatic analysis reveals the presence of viral sequences in all of the samples (Fig. 4a). Regarding the number of viral families, the most numerous are present in Bešeňová, which has the lowest water temperature. On the contrary, only two families were found in Egerszalók where water temperature is one of the highest. The Siphoviridae and Myoviridae families are the most abundant among the identified virus groups. The viruses belonging to the Herelleviridae and Herpesviridae families, as well as to the Mimiviridae and Baculoviridae families, are less common. All identified families contain DNA viruses. It is significant to note that most of the virus groups determined are characterized by capsids with an icosahedral symmetry and they are not surrounded by a lipid envelope (Fig. 4b). Importantly, bacteria and archaea are hosts for most of the viral families. On the other hand, the most numerous eukaryotic viruses are members of the Mimiviridae and Herpesviridae families. Lipid-enveloped eukaryotic viruses include the Herpesviridae with an icosahedral symmetry and Baculoviridae with a more complex structure.

The composition of the viral communities across the samples does vary. Therefore, the composition of the bacterial and archaeal communities was also checked for their differentiation (Fig. 4c). Generally, the metagenomic analyses reveal that the biofilms are composed chiefly of the same classes of bacteria, but with variations in their abundance. The classes Alphaproteobacteria, Actinomycetia, Bacilli, Bacteroidia and Flavobacteria, as well as Gammaproteobacteria, are dominant in all samples. Less abundant are Clostridia and Cyanophyceae. Among the archaea, Halobacteria, Methanomicrobia and Nitrososphaeria are common.

## Discussion and conclusions

The studied biofilms are eminently heterogeneous and VLPs are readily observed within them. However, it is difficult to demonstrate conclusively that they are actually viruses. Published data indicate that the presence of very fine fluorescent dots can be interpreted as the presence of VLPs^7,19^. However, it should be noted that the use of a suitable dye is very important here, as indicated in the current study. Note that angular (hexagonal) structures corresponding to the size of viruses can only be examined with TEM. Moreover, experimental studies also reveal the presence of similar angular structures^20^. It has been shown experimentally that viruses can both bind to mineral phases and influence mineral formation^16,20,21^. In addition, viruses present during calcium carbonate precipitation can affect both the particle size distribution of calcium carbonate and the formation of a metastable phase such as vaterite^20^.

The chemical composition of VLPs can also help in the attribution of a genuine origin of such particles as mineralized capsids. In particular all VLPs show a variable content of P and N that can be used as a proxy of residual original viral material (i.e., proteins and DNA), preserved in the mineralized particles^12^. The C, N and P elemental composition of the bacteriophage capsids, which comprise a protein shell and the RNA or DNA genetic material within it, has been calculated for a number of viruses and a predictable C:N:P ratio has been proposed, as a function of virus size^22^. Following this model, the N:P molar ratio of bacteriophages with sizes from 20 to 300 nm ranges from 15 to 4. This range of values is quite far from the N:P ratio reported here in the VLPs of travertine biofilms. This could indicate alteration of the original viral proteins and genetic material. This is not surprising if it is considered that the mineralization process and early diagenesis could have significantly altered the original organic matter composition of partially to fully mineralized VLPs.

Taking into account the shape, dimension and chemical composition of the VLP mineral structures observed within the travertine biofilms, they can be interpreted as mineralised viral capsids. It has already been suggested that capsids can act as mineral nucleation sites leading to the formation of initial nanoparticles of Ca-Mg-Si amorphous precipitates in hypersaline mats and in freshwater biofilms^7,12,14,15,23^. The process initiates mainly within the EPS, which contain negatively-charged carboxyl, phosphate and sulphate groups, as well as aspartic and glutamic acids, that can attract and concentrate available metal cations, notably Ca, Mg and Si (with less Al, K, S and P) which can then promote the mineral nucleation process^9,24–32^.

Precipitation of such mineral precursors can also be favoured by the pH increase induced by cyanobacterial photosynthesis^9^. Viral capsids themselves, have a net negative charge that can lead to the active attraction of cations, which can then promote the mineral nucleation process^10,11,16,33^, as also shown in the laboratory experiments of Ref.^20^, that reported coalesced nanospheroidal precipitates of CaCO_3_ mineralising viral capsids. At pH 6.5–8 which covers all the discussed travertine sites, the ζ potential of bacteriophages used to measure the proton-virus interactions, ranges from ~ − 4 to − 25 mV^10,16^; this confirms that virus surfaces accumulate a negative charge. The process of mineral formation is subsequently triggered by the initial mineralized amorphous nanoparticles acting as nucleation sites for the subsequent formation of both crystalline carbonates and clay minerals, during further degradation of the EPS^7,12,14,15^.

Metagenomic studies reveal that most of the virus families found in the samples are non-enveloped bacteriophages with icosahedral-shaped capsids. Two virus families, Siphoviridae and Myoviridae (Caudovirales), account for the majority of identified bacteriophages. It should be noted, however, that the taxonomy of viruses is currently undergoing very dynamic changes^34^. However, these changes will be so significant that it is currently not yet possible to implement them due to the lack of revised databases for bioinformatic analysis. The problem is serious because the classification of viruses, previously based on morphology, has completely changed. Now viral taxonomy is based on genome-level relationships. However, it seems to be of less importance for the conclusions in the present study. Considering the previous principles of viral taxonomy, the results obtained here are similar to those of Ref.^7^ from biofilms in the hypersaline environment of Lake Lagoa Vermelha (Brazil). Therefore, it is interesting to speculate whether the dominance of two bacteriophage families is a general rule. Studies of the global population of viruses show that the main families among annotated known sequences are Myoviridae, Podoviridae and Siphoviridae^35^. Here, it has been noted that the biofilms associated with the various travertine deposits are different in terms of their viral composition. But is this related to the obviously different morphology of the biofilm structure (as shown by microscopic analyses), or to more subtle differences in microbial composition as revealed by metagenomic studies? Considering the viral, bacterial and archaeal communities separately, a simple cluster analysis shows that the samples group in a quite similar fashion (Supplemental materials, Fig. S4). For instance, the Bešeňová sample often forms a separate group. From the archaeal and viral compositions, Egerszalók, Asinello and Bullicame samples group together. However, considering the whole bacterial community, four main clusters can be recognised: (i) Egerszalók, (ii) Asinello, (iii) Bullicame with Karahayit and (iv) Bešeňová, Bath, Pamukkale and Terme di Saturnia. It should be noted, that within the last cluster Bešeňová groups separately. This suggests that these compositions are interrelated which may seem obvious. However, the issue is not clear although all bacterial groups can be controlled by temperature. In the case of viruses, most of the viral sequences (even more than 90%) found in the samples remain unknown^35,36^. Moreover, the influence of external factors on the studied environments of travertine formation, including human pressure and possible pollutants, should be considered. The question of whether specific biofilm environments associated with modern carbonate deposits have their own characteristic viral communities remains to be resolved in future studies. Desnues et al.^36^ pointed out that, on the one hand, viruses are characterized by a cosmopolitan distribution; on the other hand, analysis of unknown viral sequences isolated from stromatolites showed that some phage genotypes were constrained to specific environments.

Finally, in some bacteria, the formation of minerals has been reported to be likely controlled by specific genes^37^, and the properties of newly formed organominerals are linked with their biological functions^38^. Although the genomic approach is still ‘work in progress’ it seems that new technologies, e.g. based on CRISPR-Cas systems^39^ could help to better understand the organomineralization process. Likewise, it is also plausible that in the case of viruses, capsids composed by hexameric and pentameric capsomers formed by the protein gp13 and gp12, may play an adhesion role in bacteriophage SPP1^40^. These proteins could adsorb the phage particles onto surfaces and hypothetically attract various cations to promote mineral nucleation. This mechanism, however, needs to be further explored. Therefore, the new findings reported herein add more to the understanding of the organomineralization process of bacteriophages in Ca-supersaturated thermal waters (confirmed by high values of LIS_CAL_, Table S3) and open up a new window for establishing additional precipitation models in such settings.

## Methods

### Sample collection

Biofilms associated with the travertine surfaces of deposition have been collected at the Asinello (Italy), Bath (United Kingdom), Bešeňová (Slovakia), Bullicame (Italy), Egerszalók (Hungary), Karahayıt ‘Kızılsu’ (Turkey), Pamukkale (Turkey), Terme di Saturnia (Italy) and Zitelle (Italy) sites (Supplemental materials, Table S1). Biofilm samples were placed in sterile 15 mL Falcon tubes and filled with their original water. Temperature and pH were measured directly at each site while collecting samples.

### Epifluorescence microscope

All samples were examined without fixation. For fluorescence microscopy the SYBR^®^ Green, SYBR^®^ Gold and SYPRO Tangerine were used. These stains interact with DNA, DNA/RNA and proteins respectively. Samples were put into PCR-test tubes with appropriate stain (20 μL) and diluted with 10 μL of deionized water. The staining was conducted for 15 min. The epifluorescence microscope with an HBO lamp was used to obtain the images (blue filter, DM500 with a band-pass 460–490 nm excitation filter).

### Sample preparation for transmission electron microscopy

All samples after fixation were post-fixed in 3% glutaraldehyde in their original water, dehydrated in graded acetone solutions and embedded in Araldite epoxy resin (Fluka, Buchs, Switzerland). Some samples were post-fixed in osmium tetroxide (OsO_4_) (1%) before dehydration to increase the contrast during observations; however, as some artefacts were detected due to this procedure the same samples were processes without OsO_4_. Ultrathin sections for TEM, ca 90 nm thick, were prepared using a glass knife, collected on copper grids (G 300 Cu).

### Transmission electron microscopy

TEM observations were performed with a JEOL JEM 1400 Plus instrument operating at 80 kV (JEOL Limited, Tokyo, Japan). EDS analysis was performed in the STEM mode at 80 kV accelerating voltage. Selected area diffraction (SAED) patterns were recorded using a selected area aperture 600 nm in diameter. High-resolution elemental mapping was performed on the same thin sections used for HRTEM. The experiments were carried out using a ThermoFisher Scientific (TFS, former FEI) Themis Z 80–300 microscope operated at 300 kV in scanning mode. A SuperX Energy Dispersive X-ray spectrometer, and a Gatan Imaging Filter (GIF) Continuum 1065ER were used to visualize distribution of chemical elements with a focus on nitrogen and phosphorus. The dispersion of 5 eV/ch with a total range of 20 keV was used for EDX spectroscopy.

### Metagenomic sequencing

The genomic DNA from the environmental samples was isolated using EURx kit for complex matrix (Soil DNA Purification Kit, no E3570, EURX Ltd. Poland) according to the protocol of the manufacturer. The protocol assumes the mechanical homogenisation of the samples to release the cells from the rock matrix. The isolated genomic DNA was subjected to metagenomic analysis in Genomed S.A. (Warsaw, Poland). DNA samples were mechanically fragmented with Focused-ultrasonicator Covaris E220. Libraries were prepared with NEBNext^®^ Ultra™ II DNA Library Prep Kit for Illumina® (New England Biolabs, E7645L) according to manufacturer protocol. Sequencing was performed on an Illumina NovaSeq 6000 system with paired end technology, 2 × 150nt. A 2 × greater read depth (80 million pairs instead of the standard 40 million pairs) was used to facilitate detection of viral reads. The raw sequences were pre-processed using fastp (v. 0.23.2) in order to enhance the quality of the raw reads^41^. Subsequently, the raw sequences were de novo assembled using MEGAHIT (v. 1.2.9)^42^. The minimum contig length was set at 500 bp. Subsequently, kraken2^43^ software was used for the taxonomic classification of the pre-processed raw sequences and contigs. The protocol of Ref.^44^ was used for the proper classification with kraken2 and the PlusPF database (release date 2022–09-04) was used. Additionally, species abundance estimation in samples was performed using bracken^45^ with the threshold level set at 5 (all matches less than 5 reads were discarded). Significantly, contigs were used to classify viral sequences, as the short-read approach may not be sufficient for the analysis of diverse viral sequences^46^. Some of the abovementioned analyses were performed on the Galaxy web platform (usegalaxy.eu)^47^. Before hierarchical analyses, metagenomic results were imputed to solve the problem of zero-values and subsequently were transformed with centered log-ratio, due to its compositional nature using zCompositions package for R^48,49^. The heatmaps with clustering were conducted with heatmapper using Euclidean distances^50^.

## Supplementary Information

Supplementary Information.

## Data Availability

The datasets generated during and/or analysed during the current study are available in the SRA repository, https://www.ncbi.nlm.nih.gov/sra/PRJNA957956.
