# Supplementary material for: Viruses participate in the organomineralization of travertines
Source: Sci Rep. 2023 Jul 19;13:11663. doi: 10.1038/s41598-023-38873-5 (PMC10356913; doi:10.1038/s41598-023-38873-5)
Supplement: Supplementary file 1 — Supplementary Information. [file 41598_2023_38873_MOESM1_ESM.docx]

**Viruses participate in the organomineralization of travertines**

Mirosław Słowakiewicz^a,*^, Edoardo Perri^b^, Ezher Tagliasacchi^c^, Paweł Działak^d^, Andrzej Borkowski^d^, Michał Gradziński^e^, Sándor Kele^f,g^, Maurice E. Tucker^h^

^a^Faculty of Geology, University of Warsaw, 02-089 Warsaw, Poland

^b^Dipartimento di Biologia Ecologia e Scienze della Terra, Università della Calabria, 87036 Rende, Italy

^c^Faculty of Engineering, Pamukkale University, 20160 Denizli, Turkey

^d^Faculty of Geology, Geophysics and Environmental Protection, AGH University of Science and Technology, 30-059 Kraków, Poland

^e^Institute of Geological Sciences, Jagiellonian University, 30-387 Kraków, Poland

^f^Institute for Geological and Geochemical Research, Research Centre for Astronomy and Earth Sciences, 1112 Budapest, Hungary

^g^CSFK, MTA Centre of Excellence, 1121 Budapest, Hungary

^h^School of Earth Sciences, University of Bristol, Bristol BS8 1RJ, UK

Table S1. Location, coordinates, type and mineralogy of the studied samples.

| **Location** | **Country** | **Coordinates** | **Type of samples** | **Mineralogy of carbonate deposits** |
| --- | --- | --- | --- | --- |
| Egerszalók travertine | Hungary | 47°51'11.95"N, 20°20'02.83"E | Green biofilm samples from distal part of system, collected below and farther from mound | Calcite, aragonite (^1^) |
| Sacred Spring travertine (Bath) | UK | 51°22'50.67"N, 2°21'34.60"W | Biofilm with precipitates close to spring | Calcite, ferrihydrite |
| Great Bath travertine (Bath) | UK | 51°22'50.67"N, 2°21'34.60"W | Biofilm with precipitates close to travertine | Calcite, ferrihydrite |
| Terme di Saturnia travertine | Italy | 42°39'31.23"N, 11°30'59.61"E | Biofilm on surface of travertine, distal part of spring and close to pools | Calcite (^2^) |
| Bullicame travertine | Italy | 42°25'12.98"N, 12°04'22.07"E | Biofilm within travertine, close to hot spring | Calcite, aragonite (^3,2^) |
| Asinello travertine | Italy | 42°23'57.01"N, 12° 3'31.71"E | Biofilm with travertine close to spring | Calcite, probable aragonite (no data) |
| Zitelle travertine | Italy | 42° 25' 32.73"N, 12° 3' 40.75"E | Biofilm with travertine collected close to hot spring | Aragonite, calcite (^4^) |
| Karahayıt ‘Kızılsu’ travertine | Turkey | 37°58'2.42"N, 29°6'9.30"E | Biofilm on surface of precipitates, close to hot spring | Calcite, aragonite (^5^) |
| Bešeňová travertine | Slovakia | 49°06'13.83"N, 19°26'09.95"E | Brown biofilm attached to travertine, 4 cm from spring, in channel over travertine cascade | Calcite, goethite |
| Pamukkale travertine | Turkey | 37°55'24.46"N, 29°07'23.28"E | Biofilm on surface of precipitates, samples collected from channel farther from spring | Calcite, aragonite (^5^) |

Table S2. Temperature and pH of water from the studied sampling sites.

| **Location** | **pH** | **temp** | **season** | **References** |
| --- | --- | --- | --- | --- |
| Egerszalók travertine | 6.4 | 45 | spring | ^1,6^ |
| Sacred Spring travertine (Bath) | 7.1 | 46 | spring | this study |
| Great Bath travertine (Bath) | 7.9 | 38 | spring | this study |
| Terme di Saturnia travertine | 7.5 | 33.4 | spring | ^2^ |
| Bullicame travertine | 6.64 | 51.3 | spring | ^7,3,8,2,9^ |
| Asinello travertine | 6.17 | 47.5 | spring | this study |
| Zitelle travertine | 7.19 | 51.9 | spring | ^9^ |
| Karahayıt ‘Kızılsu’ travertine | 7.2 | 36.4 | spring | ^10,5,11,12^ |
| Bešeňová travertine | 6.21 | 14.6 | spring | this study |
| Pamukkale travertine | 6.57 | 26.1 | spring | ^10,5,11–13^ |

Table S3. Chemical composition of water from the studied sampling sites. LSI_CAL_ – Langelier saturation index with respect to calcite.

| **Location** | Ca | Mg | Na | K | Li | Sr | Mn | Ba | Fe | HCO_3_ | SO_4_ | Cl | **Conductivity (μs/cm)** | **LSI_CAL_** | **Reference** |
| --- | --- | --- | --- | --- | --- | --- | --- | --- | --- | --- | --- | --- | --- | --- | --- |
|  | **mg L^-1^** | | | | | | | | | | | |  |  |  |
| Pamukkale | 450 | 88 | 42 | 5.7 |  | 6.37 | 70 |  | 280 | 945 | 682 | 10 | 2380 | 0.14 | ^5^ |
| Karahayıt Kızılsu | 449 | 114 | 115 | 23 |  | 9.09 |  |  |  | 947 | 961 | 26.9 | 2480 | 0.96 | ^5^ |
| Egerszalók | 160 | 24.8 | 59.5 | 14 |  | 1.6 | 0.001 | 180 | 0.02 | 630 | 81 | 23 | 990 | -0.09 | ^1^ |
| Terme di Saturnia | 593 | 92 | 45 | 6 | 503 | 10879 | 23 |  | 1.7 | 662 | 1541 | 67 | 2360 | 1.2 | ^14^ |
| Bath | 285 | 51 | 218 | 20 | 0.4 | 6600 | 46 | 32 | 774 | 187 | 1080 | 344 | 2560 | -0.41 | ^15^ |
| Asinello | 632 | 155 | 33 | 33 | 0.08 | 10.95 |  |  |  | 1056 | 1350 | 17 | 2670 | 0.32 | ^16,17^ |
| Bullicame | 510 | 110 | 34 | 27 | 3.1 | 12 | 28 |  | 12 | 990 | 1060 | 14 | 2290 | 0.77 | ^18^ |
| Bešeňová | 567 | 176.6 | 88.25 | 38.66 | 0.311 | 5.96 | 0.0578 | 0.02 | 0.2255 | 1851 | 822 | 23.63 | 3220 | -0.15 | this study |
| Zitelle | 580 | 130 | 36 | 38 | 130 | 11 | 22 |  | 8.5 | 1000 | 1300 | 13 | 2300 | 1.4 | ^18^ |


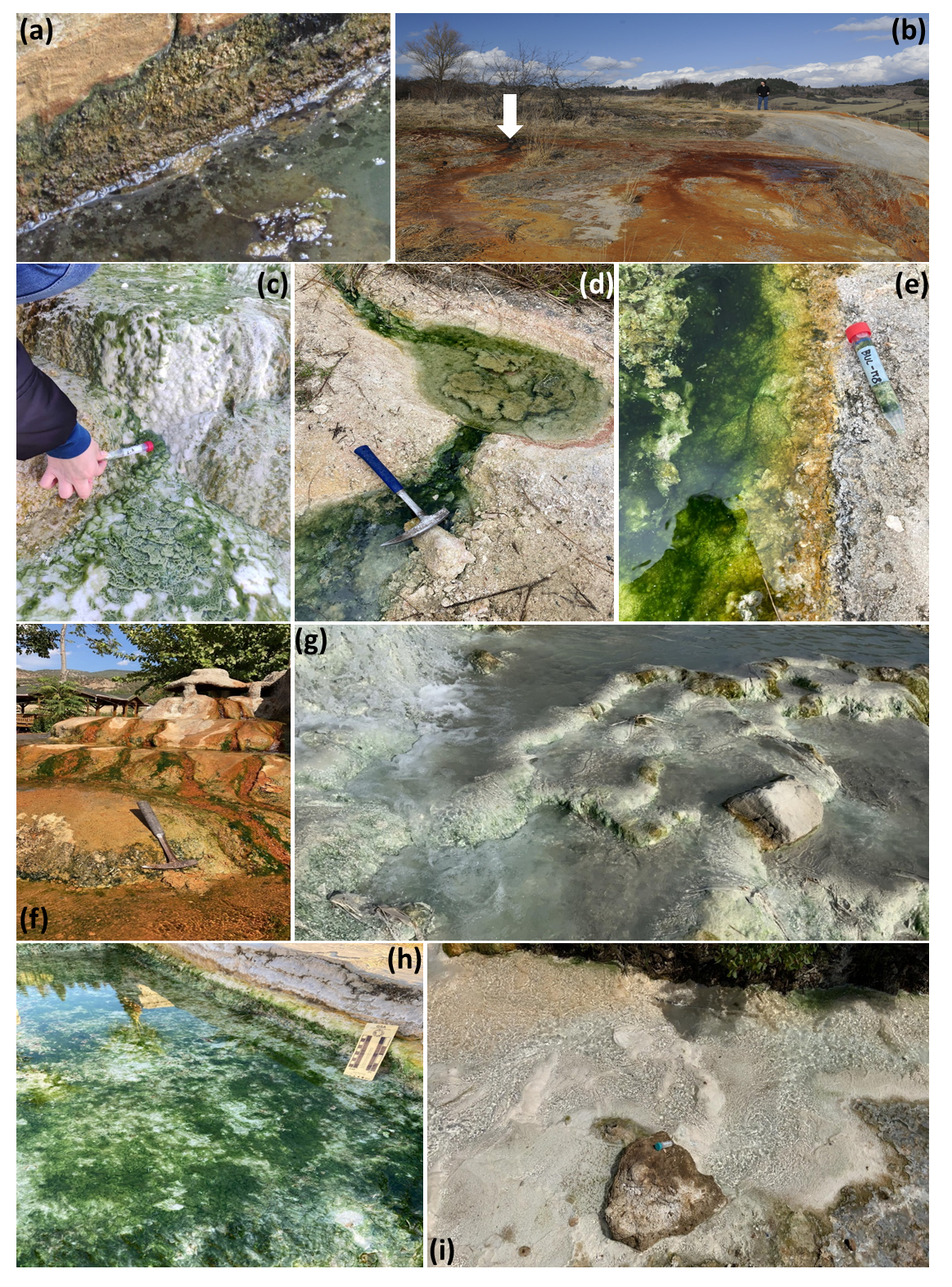


Fig. S1. Images of the studied biofilm samples at the mesoscale. (a) Cindery travertine precipitated near base of Roman pillar from biofilm at and below water level; also floating biofilm with air-bubbles, within which calcite and ferrihydrite are being precipitated. The Great Bath, Bath, England; field of view 40 cm across; (b) Sampling site (arrow) at Bešeňová, travertine cascade to the right. Biofilms with brownish colour. Person for scale 180 cm tall; (c) Green biofilm collected at the distal parts of the Egerszalók travertine mound, below the cascades and approximately 25-30 m away from the thermal well. Falcon tubes in this and other images are 12 cm long; (d, e, f, g) Green biofilms growing on the surface of travertines at Asinello, Bullicame, Karahayıt Kızılsu (biofilms close to the thermal spring), and Terme di Saturnia (a pool and rim in distal part), respectively. Hammer 28 cm long. Field of view in (g) 2 m across; (h) Green biofilms on the surface and interlayered with travertine at Pamukkale. Scale is 10 cm; (i) Brown to green biofilms on the surface of Zitelle travertine.


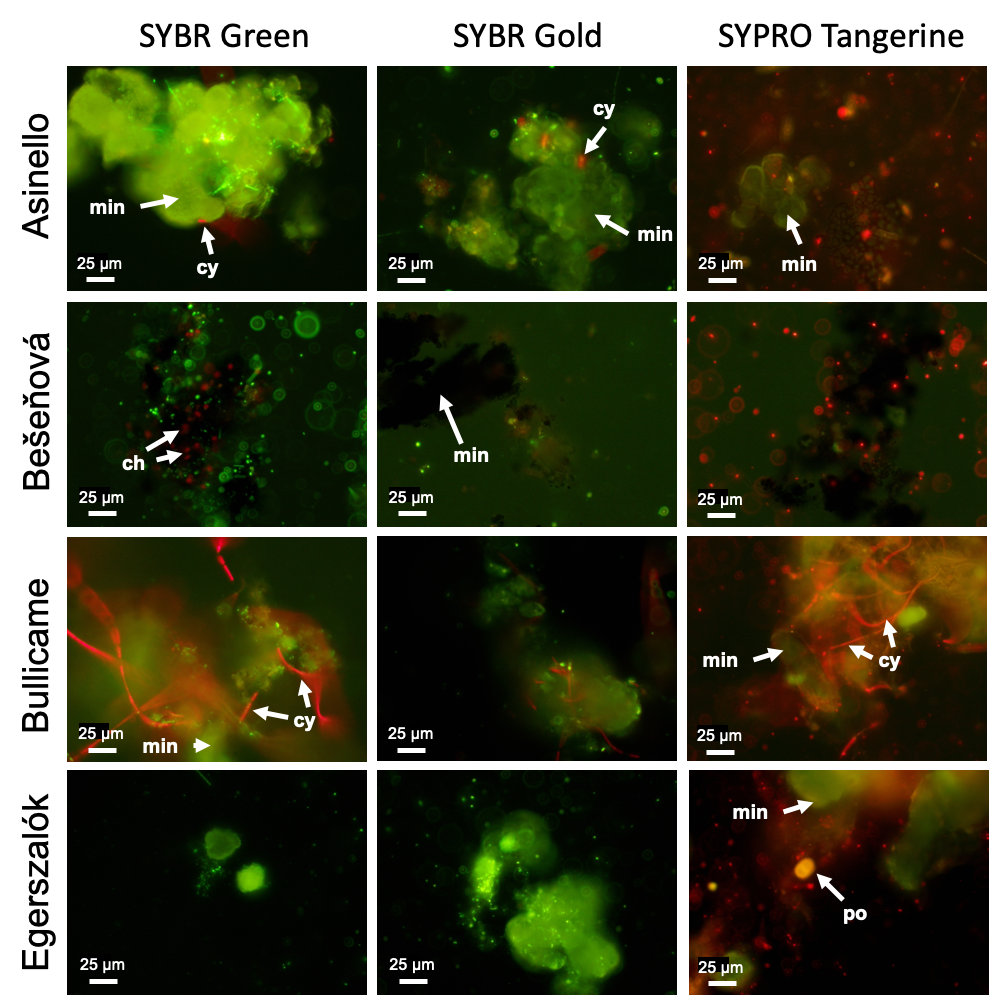


Fig. S2. Samples of biofilms under epifluorescence microscope. The fluorescence stains are indicated: SYBR Green, SYBR Gold and SYPRO Tangerine. Examples of visible microorganisms and particles marked: cy – cyanobacteria; po – pollen; min – mineral phases; ch – fluorescence of chlorophyll from cyanobacteria or algae. Green or red dots (dependent on the stain used) are microorganisms. Virus-like particles are potentially visible as the smallest green dots only after SYBR Gold staining.


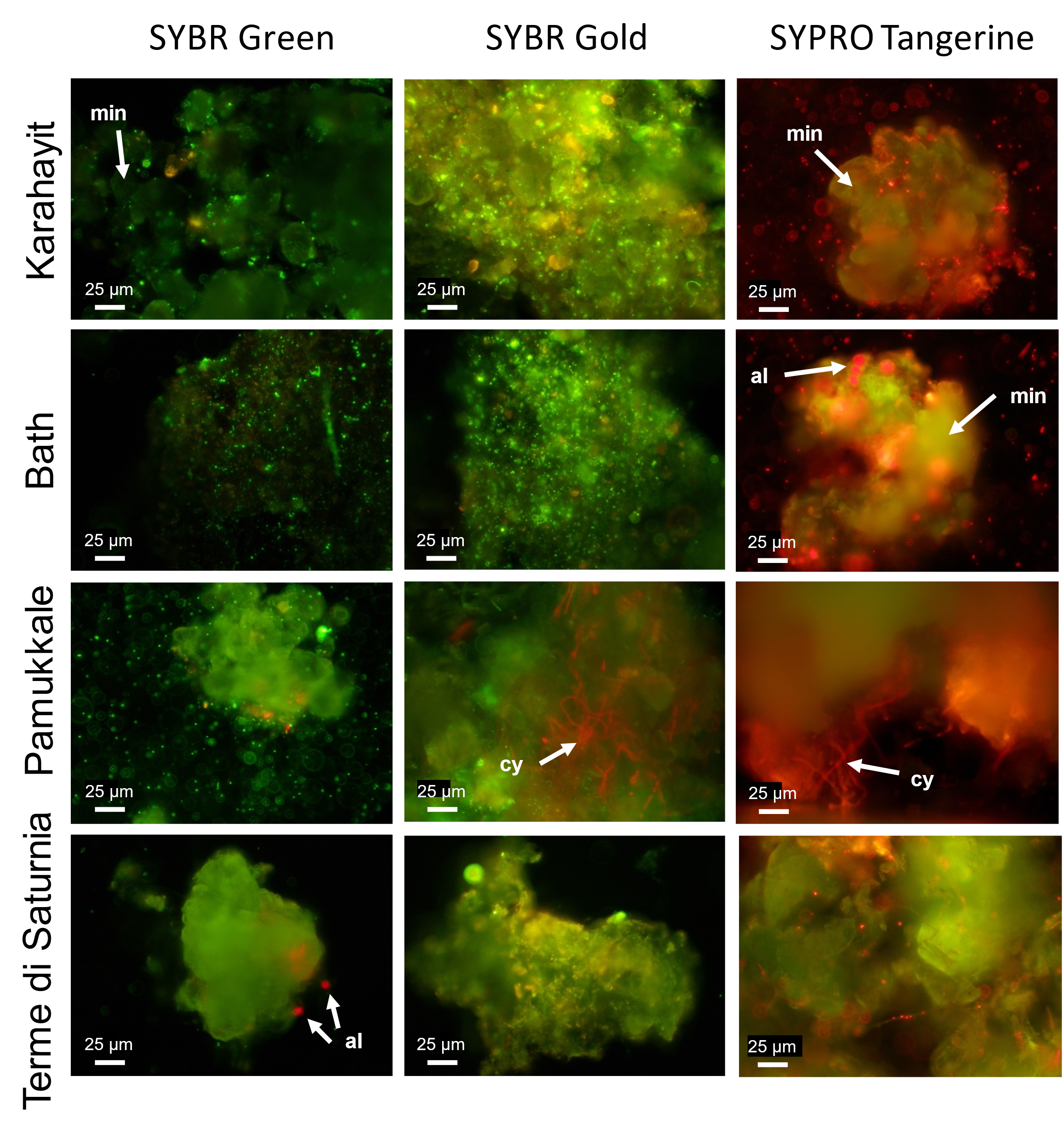


Fig. S3. Samples of biofilms under epifluorescence microscope. The fluorescence stains are indicated: SYBR Green, SYBR Gold and SYPRO Tangerine. Examples of visible microorganisms and particles marked: cy – cyanobacteria; min – mineral phases. Green or red dots (dependent on the stain used) are microorganisms. Virus-like particles are potentially visible as the smallest green dots only after SYBR Gold staining.


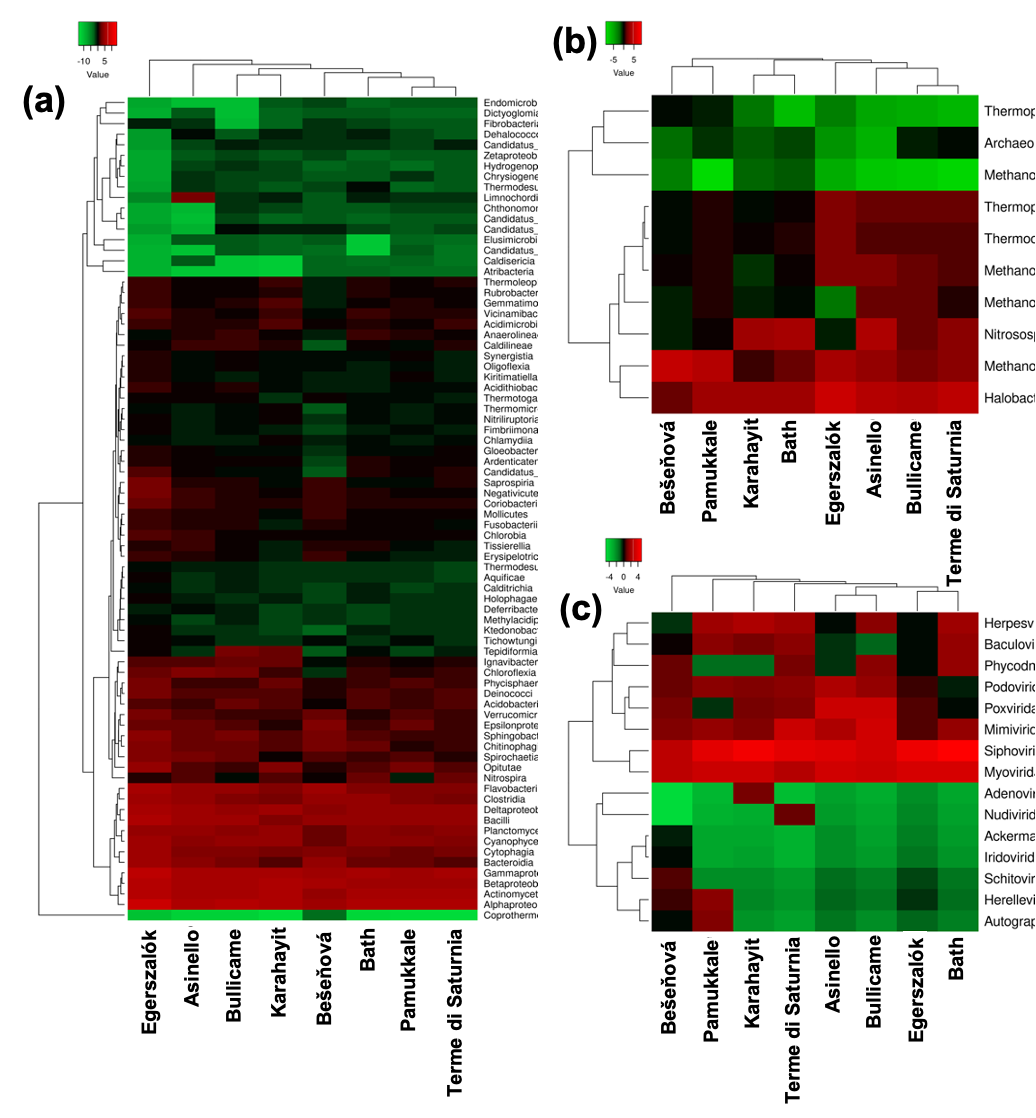


Fig. S4. Hierarchical analyses with heatmaps of samples dependent on (a) bacterial community, (b) archaeal community and (c) viral composition. Zero-values are imputed and data are transformed with Centered log-ratio.

**References**

1. Kele, S. *et al.* Chemical and stable isotope composition of recent hot-water travertines and associated thermal waters, from Egerszalók, Hungary: Depositional facies and non-equilibrium fractionation. *Sedimentary Geology* **211**, 53–72 (2008).

2. Della Porta, G., Hoppert, M., Hallmann, C., Schneider, D. & Reitner, J. The influence of microbial mats on travertine precipitation in active hydrothermal systems (Central Italy). *The Depositional Record* **8**, 165–209 (2022).

3. Di Benedetto, F. *et al.* Biotic and inorganic control on travertine deposition at Bullicame 3 spring (Viterbo, Italy): A multidisciplinary approach. *Geochimica et Cosmochimica Acta* **75**, 4441–4455 (2011).

4. Folk, R. Interaction between bacteria, nannobacteria and mineral precipitation in hot springs of Central Italy. *Géographie physique et Quaternaire* **48**, 233–246 (1994).

5. Özkul, M. *et al.* Comparison of the Quaternary travertine sites in the Denizli extensional basin based on their depositional and geochemical data. *Sedimentary Geology* **294**, 179–204 (2013).

6. Hałaj, E. & Wachowicz-Pyzik, A. Examples of applications of geothermal waters for recreation, heating and bottling in selected regions of Hungary. *Geology, Geophysics and Environment* **39**, 21–32 (2013).

7. Piscopo, V. *et al.* Hydrogeology of thermal waters in Viterbo area, central Italy. *Hydrogeology Journal* **14**, 1508–1521 (2006).

8. Chiocchini, U. & Savarese, G. The Viterbo hydrothermal system and its sustainable exploitation, Central Italy. *Journal of Energy and Power Technology* **1**, 1–23 (2019).

9. Rimondi, V. *et al.* Bioaccessible arsenic in soil of thermal areas of Viterbo, Central Italy: implications for human health risk. *Environmental Geochemistry and Health* **44**, 465–485 (2022).

10. Kele, S. *et al.* Stable isotope geochemical study of Pamukkale travertines: New evidences of low-temperature non-equilibrium calcite-water fractionation. *Sedimentary Geology* **238**, 191–212 (2011).

11. Özkul, M. *et al.* Sedimentological and geochemical characteristics of a fluvial travertine: A case from the eastern Mediterranean region. *Sedimentology* **61**, 291–318 (2014).

12. Alçiçek, H., Bülbül, A., Yavuzer, İ. & Cihat Alçiçek, M. Origin and evolution of the thermal waters from the Pamukkale Geothermal Field (Denizli Basin, SW Anatolia, Turkey): Insights from hydrogeochemistry and geothermometry. *Journal of Volcanology and Geothermal Research* **372**, 48–70 (2019).

13. Rizzo, A. L. *et al.* Geochemistry of fluid inclusions in travertines from Western and Northern Turkey: Inferences on the role of active faults in fluids circulation. *Geochemistry, Geophysics, Geosystems* **20**, 5473–5498 (2019).

14. Barbagli, A. *et al.* Multi-isotope and Hydrogeochemical approach for characterizing Saturnia thermal groundwater (Grosseto, Italy). *Acque Sotterranee - Italian Journal of Groundwater* **2**, (2013).

15. Edmunds, W. M., Darling, W. G., Purtschert, R. & Corcho Alvarado, J. A. Noble gas, CFC and other geochemical evidence for the age and origin of the Bath thermal waters, UK. *Applied Geochemistry* **40**, 155–163 (2014).

16. Chiocchini, U., Castaldi, F., Barbieri, M. & Eulilli, V. A stratigraphic and geophysical approach to studying the deep-circulating groundwater and thermal springs, and their recharge areas, in Cimini Mountains–Viterbo area, central Italy. *Hydrogeology Journal* **18**, 1319–1341 (2010).

17. Sensi, S. I depositi carbonatici di travertino situati nella zona l’Asinello (Viterbo). (Università degli Studi della Tuscia, 2016).

18. Cinti, D. *et al.* Fluid geochemistry and geothermometry in the unexploited geothermal field of the Vicano–Cimino Volcanic District (Central Italy). *Chemical Geology* **371**, 96–114 (2014).
